# Optimization of Its Preparation and Evaluation of Fresh and Tender Leaves of *Artemisia argyi* for Food Usage

**DOI:** 10.3390/foods14183185

**Published:** 2025-09-12

**Authors:** Xiaoyan Qin, Xujia Liu, Ping Qin, Lijuan Wang, Guoxun Chen, Fang Yang

**Affiliations:** 1 School of Laboratory Medicine, Hubei University of Chinese Medicine, Wuhan 430065, China; 2 College of Food Science and Technology, Huazhong Agricultural University, Wuhan 430070, China; 3 Hubei Institute of Qiai Industrial Technology, Huanggang 435300, China

**Keywords:** *Artemisia argyi*, alkaline boiling, acute toxicity test, food safety, caecal microbiota

## Abstract

*Artemisia argyi* (*A. argyi*), a perennial herb with a thick and intense aroma, has traditionally been consumed for over a thousand years without any side effect in many parts of China and East Asia. However, a formal way of treatment in the food industry to prepare products for its safe use in the market has not been standardized. This study developed a safe and easy process to boil fresh and tender leaves of *A. argyi* in a 1% NaHCO_3_ solution for 2 min, soak the product in cold water and dry it at or below 40 °C, which resulted in base materials for food usage. Nutritional profiling revealed that the boiled fresh and tender leaves of *A. argyi* (BAAQ) powder contained crude protein, dietary fiber, vitamins and minerals. In addition to improving the BAAQ powder’s organoleptic qualities, alkaline boiling and soaking processes significantly reduced some potentially harmful or irritating substances. The safety of BAAQ powder was evaluated using rats. It showed practically non-toxicity in acute oral toxicity test (LD_50_ > 7.5 g/kg BW). The treatment of BAAQ powder modulated the composition of the caecal microbiota in rats by enhancing beneficial taxonomic groups (*Lactobacillus*, *Oscillibacter*, and *Ruminococcus*) and inhibiting opportunistic pathogenic bacteria (*Enterococcus* and *Staphylococcus*). This study delineates the optimal harvesting season, edible portions of *A. argyi*, and processing techniques for its safe use. It ensures the safety of foods derived from BAAQ powder and initiates the industrialization of *A. argyi* foods.

## 1. Introduction

*Artemisia argyi* H. Lév. & Vaniot (*A. argyi*), commonly known as mugwort or moxa, is a perennial herb of genus *Artemisia* in the Asteraceae family, with a thick and intense aroma. It is widely distributed in the moderate climate zones of East Asia, Europe and North America [1]. This traditional medicinal and culinary plant is referred to as “Aicao” in Chinese, “Aeyup” in Korean and “Gaiyou” or “Yomogi” in Japanese [2]. Traditionally, the tender leaves of *A. argyi* are consumed as a vegetable, tea or functional ingredient in traditional foods such as “Qingtuan” in China, “Yomogi-mochi” in Japan, and various soups and beverages in Korea [3]. The consumption history of *A. argyi* can be traced back to the Tang Dynasty in China. Through the investigation of eating history, we have found that residents in Qichun County of Hubei Province and its surrounding areas have the habit of eating *A. argyi* in traditional ways. The average eating time of *A. argyi* is more than 30 years, and the daily consumption is about 9 g [4]. The edible forms of *A. argyi* mainly include fresh leaves, pastries, noodles, tea brewing, medicinal use and wine brewing, and no negative reactions have been reported [4]. In addition to the use of its fresh and tender leaves as a dietary ingredient, *A. argyi* is also utilized in various traditional medicinal practices. Mature leaves and stalks are often employed in moxibustion therapy, while dried aged leaves are used in medicinal sachets believed to offer health benefits through aromatic release [3]. Chinese people also take bath in water prepared from the boiled *A. argyi* leaves.

In the *Flora of China* and the *China Food Composition Table*, *A. argyi* is classified as an edible wild vegetable, with its buds, tender leaves and seedlings being the edible parts [5,6,7]. The fresh and tender leaves of *A. argyi* are harvested at the vegetative growth stage, before flowering, typically around April, corresponding to the traditional harvesting period around Qingming Festival or Tomb-Sweeping Day (April 4th or 5th). It is often processed using an alkaline boiling method, which involves cooking the leaves in a weak alkaline solution to reduce bitterness, enhance green color and improve texture. Because of their bright green color, unique aromas and health benefits, the fresh and tender leaves of *A. argyi* are widely consumed as a traditional food ingredient and tea in East Asia. The leaves are rich in dietary fiber, vitamins, flavonoids, polyphenols and trace elements [8]. The bioactive constituents derived from *A. argyi* include volatile oil, flavonoids, polysaccharides, phenolic acids, terpenoids, alkaloids, esters, lignans and coumarins, quinones and tannins [8,9,10]. These products and active ingredients from *A. argyi* have shown excellent antibacterial [11], antiviral [12], anti-inflammatory [2,13], antioxidant [8], anti-osteoporosis, anticoagulant [14] and anti-tumor activities [10], immune function enhancement [14,15,16], nerve protection [17,18] and other bioactivities.

The food resources of China are abundant and diverse with a rich history. Nevertheless, a large number of safe and reliable food resources, especially some foods with traditional and local characteristics, remain to be introduced to the market by the food industry. Given its rich history, *A. argyi* is a candidate for the potential future food resources. The traditional preparation of the tender leaves of *A. argyi* for making the food “Qingtuan” requires the treatment of the leaves in boiling water with NaHCO_3_ (alkaline water) and soaking in cold water to eliminate volatile components and keep their vibrant green and aroma. Toxicological studies have mostly focused on medicinal, dried and mature leaves or their volatile oils [19], ignoring the safety of the edible portions and how they are prepared using conventional culinary techniques. Therefore, with reference to this traditional process, a procedure was developed to produce a safe and nutritious food ingredient from the tender leaves of *A. argyi*. This study optimized the preparation procedure for fresh and tender leaves of *A. argyi* and investigated its safety and regulatory effects on intestinal microecology in rats.

## 2. Materials and Methods

### 2.1. Material

The fresh and tender leaves of *A. argyi* cv. Qiai (AAQ) were provided by Hubei Institute of Qiai Industrial Technology and refrigerated or stored at −20 °C upon arrival if not treated immediately. The leaves were harvested during the vegetative growth stage prior to flowering, typically corresponding to the early spring period (March to April), from the Huayuangang planting base (35°0′34″ N; 113°5′10″ E) in Qichun County (Hubei, China). The plant was identified by Professor Dingrong Wan in the College of Pharmacy at South-Central Minzu University. Rutin (UV ≥ 97%), gallic acid (HPLC ≥ 98%), α-thujone (GC ≥ 98%), camphor (GC ≥ 98%), vitamin B_2_ (HPLC ≥ 99%), vitamin C (HPLC ≥ 99%) and β –carotene (HPLC ≥ 98%) were purchased from Shanghai Yuanye Biotechnology Co., Ltd. (Shanghai, China). Borneol (GC > 98%) and eucalyptol (GC > 99.5%) were obtained from Shanghai Meryl Biochemical Technology Co., Ltd. (Shanghai, China). Alanine aminotransferase (ALT), aspartate aminotransferase (AST), total protein (TP), albumin (ALB), alkaline phosphatase (ALP), gamma-glutamyl transferase (GGT), glucose (GLU), blood urea nitrogen (BUN), creatinine (CREA), total cholesterol (TC), triglycerides (TG), high-density lipoprotein cholesterol (HDL-C) and low-density lipoprotein cholesterol (LDL-C) kits were purchased from Nanjing Jiancheng Bioengineering Institute (Nanjing, China). The bacterial genomic DNA extraction kit and two percent agarose gel were purchased from Beijing Solarbio Science & Technology Co., Ltd. (Beijing, China). Primers were synthesized by Sangon Biotech (Shanghai, China). New England Biolabs provided the Phusion High-Fidelity PCR Master Mix with GC Buffer and DNA polymerase. Universal DNA Purification Kit was purchased from TianGen Biochemical Technology. The TruSeq^®^ DNA library preparation kit and Illumina sequencing services were obtained from Illumina (Illumina, CA, USA). All chemicals and reagents utilized were of analytical grade, and ultrapure water was employed consistently throughout the study.

### 2.2. Preparation and Optimization of Edible Raw Materials Derived from the Tender Leaves of A. argyi

**Figure 1:**
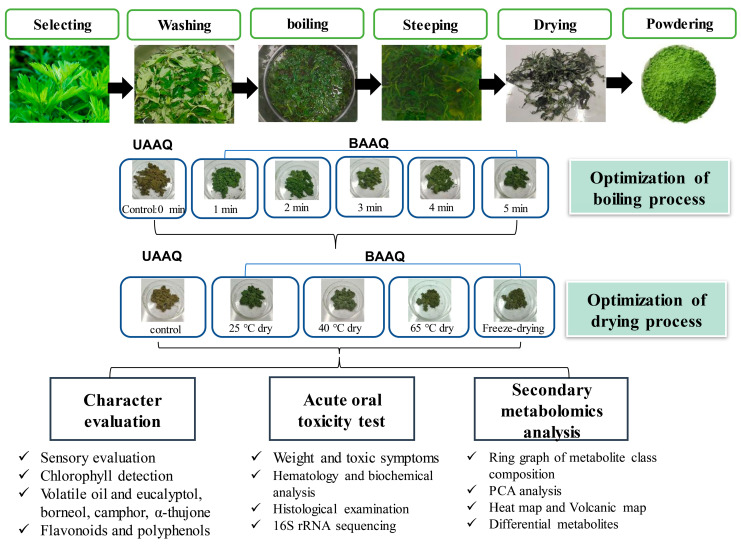
Preparation and optimization of edible raw materials derived from tender leaves of *A. argyi*. BAAQ, boiled tender leaves of *A. argyi* cv. Qiai; UAAQ, untreated tender leaves of *A. argyi* cv. Qiai.

The powder of boiled fresh and tender leaves of AAQ (BAAQ) was prepared by referring to the traditional process of making an ingredient in food “Qingtuan”. In early spring, the fresh and tender leaves at the top of *A. argyi* (5–10 cm) were picked and selected to remove weeds, branches and moldy and rotten leaves. Then, the tender leaves were weighed, washed and added to a boiling 1% NaHCO_3_ solution in a ratio of 1:10 *w*/*v* for 1–5 min. Subsequently, the boiled leaves were soaked in over 10-fold cold water for 30 min to eliminate the bitter and astringent taste. The leaves were dehydrated at 500 × g for 30 s to remove excess water using a centrifugal dehydrator (Diameter 1000, Shandong Xuxin Machinery Co., Ltd., Yantai, China). Afterward, the dehydrated leaves were dried, ground (RT-04, Zhejiang Hongjingtian Industry & Trade Co., Ltd., Wuyi, China) and sieved through an 80 mesh sieve to get the final powder for storage at −80 °C. The drying process employed hot air-drying (25 °C, 40 °C, 65 °C) or freeze-drying using a freeze dryer (SCIENTZ-10N, Ningbo Scientz Biotechnology Co., Ltd., Ningbo, China) at a condenser temperature of −60 °C and a vacuum level below 10 Pa for 24 h. Optimizations of the boiling and drying conditions were conducted, as shown in Figure 1. The basis for choosing the optimal boiling and drying conditions was to retain the bioactive compounds (especially total flavonoids and polyphenols) to the greatest extent, while ensuring the ideal physical and sensory properties of the final product. The untreated fresh and tender leaves of AAQ (UAAQ) as the control were picked, selected, washed, freeze-dried, ground and sieved to obtain leaf powder and stored at −80 °C.

### 2.3. Characteristic Evaluation

#### 2.3.1. Sensory Evaluation

Fifteen trained panelists were recruited to assess the quality characteristics of *A. argyi* leaves and their processed products in a sensory evaluation study [8]. The panelists were selected based on prior experience in descriptive sensory analysis and received additional training to identify and quantify sensory attributes specific to *A. argyi* products. The evaluation employed Quantitative Descriptive Analysis (QDA) [20], in which the panelists rated the intensity of key sensory attributes that included appearance color, aroma intensity, bitterness intensity, and bitterness persistence using a structured intensity scale (Table S1). All assessments were performed under controlled conditions in individual sensory booths. The samples were systematically evaluated in the final AAQ powder obtained from the processing method. This multi-stage, multi-form evaluation approach allowed for an in-depth comparison of sensory properties from the raw material to the final product throughout the processing stages.

#### 2.3.2. Chlorophyll Detection

The chlorophyll content was quantitatively analyzed using a spectrophotometric method. Briefly, 0.1 g of AAQ powder was homogenized with a mortar and pestle in 3 mL of 95% ethanol containing 0.1 g of calcium carbonate (as a stabilizer) [21]. The homogenate was incubated at room temperature for 3–5 min to facilitate pigment extraction. To ensure complete extraction, the residue was rinsed three times with 2 mL 95% ethanol, and the combined extracts were adjusted to a volume of 10 mL with 95% ethanol. Each sample was analyzed in triplicate. Absorbance measurements were conducted using a UV-visible spectrophotometer (V-1000, AOE Instruments (Shanghai) Co., Ltd., Shanghai, China). The absorbance of each sample was recorded at 663 nm and 645 nm, with 95% ethanol serving as the blank reference [22]. The total chlorophyll content was subsequently calculated using the following Equations (1)–(3), which were adapted accordingly to accommodate the wavelength shift.

Chla = 13.95A_663_ − 6.8A_645_ (1)

Chlb = 24.96A_645_ − 7.32A_663_ (2)

Total Chl = 18.16A_645_ + 6.63A_663_ (3)

#### 2.3.3. Extraction and Determination of Flavonoids and Polyphenols

The analysis of bioactive compounds, including flavonoids and polyphenols, in AAQ powder was conducted utilizing standardized methods [23]. In the process of ethanol extraction, 1 g of AAQ powder was mixed with a 60% ethanol solution at a solid-to-liquid ratio of 1:40 (g/mL). The mixture was then subjected to ultrasonic-assisted extraction for 45 min at 65 °C and a frequency of 40 kHz using an ultrasonic device (JTL-600, Jietuo Ultrasonic Equipment Co., Ltd., Shenzhen, China). Following centrifugation at 3600× *g* for 5 min at room temperature, the resulting supernatant was utilized as the crude flavonoids and polyphenols extract.

The quantification of flavonoids was conducted using a UV-visible spectrophotometer [24]. In brief, 1.0 mL of ethanol extract was combined with 0.3 mL of 5% NaNO_2_, 0.3 mL of 10% Al(NO_3_)_3_ and 4.0 mL of 4% NaOH. Then, the mixture was diluted with purified water to a final volume of 10 mL, and its absorbance at 510-nm wavelength was measured. The Folin–Ciocâlteu assay was employed to determine the polyphenol content [25]. Here, 1.0 mL of ethanol extract was mixed with 5.0 mL of 10% Folin–Ciocâlteu reagent for 8 min, and subsequently neutralized using 4 mL of 7.5% Na_2_CO_3_, and the absorbance was recorded at 765 nm wavelength. All analyses were performed in triplicate, and quantification was carried out using calibration curves based on rutin and gallic acid equivalents for flavonoid and polyphenol contents, respectively.

#### 2.3.4. Volatile Oil Content and Composition Analysis

The volatile oil contents of AAQ powder were measured in accordance with the procedure outline in the Pharmacopoeia of the People’s Republic of China (2020 edition) by a volatile oil determination equipment with steam distillation method. Briefly, 300 g of AAQ powder was soaked in 3 L of purified water (1:10, *w*/*v*) for 2 h, then the mixture was loaded into the volatile oil determination apparatus (Shanghai Leigu Instrument Co., Ltd., Shanghai, China) and distilled for 5 h until the volatile oil volume no longer increased. The extracted volatile oil was dehydrated with anhydrous Na_2_SO_4_ and stored at 4 °C. The extraction yields of volatile oil was calculated using the following Formula (4).

(4) $$Extraction\;yield=\frac{Weight\;of\;volatile\;oil}{Weight\;of\;powders}\times 100$$

The contents of eucalyptol, borneol, camphor and α-thujone in the volatile oil were analyzed with GC-2010 Pro gas chromatograph (Shimadzu Corporation, Kyoto, Japan) in accordance with General Chapter 0521 of the Pharmacopoeia of the People’s Republic of China (2020 Edition). The operation was performed using the following conditions: N_2_ at 30 mL·min^−1^, H_2_ at 40 mL·min^−1^, air at 400 mL·min^−1^; split ratio 5:1; injection temperature 240 °C, detector temperature 250 °C. Chromatographic column was an Agilent HP-5 capillary column (30 m × 0.32 mm × 0.25 μm; Agilent Technologies, Santa Clara, CA, USA). Column temperature programmed initialized from 45 °C to 75 °C at 2 °C·min^−1^ and held for 5 min, ramped to 90 °C at 1 °C·min^−1^ and maintained for 6 min, then increased to 150 °C at 5 °C·min^−1^, and finally raised to 250 °C at 10 °C·min^−1^ and held for 5 min. Both the reference standard solution and test solution (1 μL each) were injected in triplicate. Compounds were identified using their reference standard and retention time, and quantified through their calibration curve derived from peak areas.

### 2.4. Nutritional Composition Analysis

The moisture content in BAAQ powder was assessed following the guidelines in the Chinese National Food Safety Standard of moisture in food (GB5009.3-2016) [26]. The analysis of ash, crude protein and crude fat contents was conducted employing Method 1 of GB 5009.4-2016 [27], Method 1 of GB 5009.5-2016 [28] and Method 2 of GB 5009.6-2016 [29], respectively. The crude fiber content was determined in accordance with GB 5009.10-2003 [30]. The quantification of β-carotene, Vitamin C and B_2_ was conducted utilizing Method 1, as outlined in GB 5009.83-2016 [31], GB 5009.86-2016 [32] and GB 5009.85-2016 [33], respectively. Selenium, copper, aluminum, zinc, iron, calcium, potassium, sodium and manganese were analyzed by inductively coupled plasma mass spectrometry (ICP-MS) (Agilent 7900, Agilent Technologies, Santa Clara, CA, USA) according to ISO 17294-2:2016 [34].

### 2.5. Acute Oral Toxicity Test

The acute oral toxicity study was conducted in strict accordance with the Chinese National Food Safety Standard for acute oral toxicity test (GB 15193.3-2014) [35]. Sixty 8-week-old Wistar rats (180–220 g), 50% males and 50% females, were obtained from Hubei Provincial Laboratory Animal Research Center with the production allowance number SCXK 2020-0018. All the rats were kept in a specific pathogen-free (SPF) environment that had a consistent temperature (23 ± 2 °C), a 12 h dark–light cycle, and steady moisture (50 ± 5%). All animals received the maintenance diet from Wanqian Jiaxing Biotechnology Co., Ltd. (Wuhan, China). This animal experiment had passed the animal ethics review of the Food and Drug Safety Evaluation Center of Hubei Provincial Center for Disease Control and Prevention, with license number SCXK 2022-0065. After 1 week of accommodation, rats were randomly assigned into six groups, female control (FC), female UAAQ (FUAAQ), female BAAQ (FBAAQ), male control (MC), male UAAQ (MUAAQ), and male BAAQ (MBAAQ), 10 rats per group. BAAQ powder was prepared by alkaline boiling for 2 min followed by freeze-drying treatment. After fasting for 16 h, gavage administration was performed, with three doses administered within 24 h; each group except controls received a gavage dosage of 7.5 g/kg·BW AAQ powder in one day. Animals were observed daily after the administration for 14 consecutive days.

#### 2.5.1. Weight and Toxic Symptoms

Body weights were measured on the day of administration (day 1), day 8, and day 15. Toxicity signs were monitored daily for 14 days, with survival status including mortality documented [36]. Surviving animals underwent euthanasia and necropsy. Target organs (liver, spleen, kidney, testis, visceral fat) were harvested and weighed. Gross pathology was performed to examine external, thoracic/abdominal and cranial organs, with findings systematically recorded.

#### 2.5.2. Hematology and Biochemical Analysis

After 14 days, all rats were fasted for 12 h, and jugular vein blood samples (~1 mL) were collected. Hematological parameters in the blood sample, including white blood cell (WBC) count, red blood cell (RBC) count, hemoglobin (HGB), hematocrit (HCT), platelet (PLT) count, mean platelet volume (MPV), plateletcrit (PCT), mean corpuscular volume (MCV), mean corpuscular hemoglobin (MCH), mean corpuscular hemoglobin concentration (MCHC), neutrophil percentage (NEUT%), absolute lymphocyte (LYMPH) count, monocyte (MONO) count, eosinophil (EO) count and basophil (BASO) count, were detected by an automatic hematology analyzer (BC-760 CS, Mindraym, Shenzhen, China).

To obtain serum, the blood was kept at 4 °C for 1 h, then centrifuged at 1000× *g* for 20 min. The concentrations of ALT (C009-3), AST (C010-3), TP (A045-2), ALB (A028-2), ALP (A059-2), GGT (C017-2-1), GLU (F006-1-1), BUN (C013-2-1), CREA (C011-2-1), TC (A111-1-1), TG (A110-1-1), HDL-C (A112-1-1) and LDL-C (A113-1-1) in serum were measured using enzyme-linked immunosorbent assay (ELISA) kits and an automated clinical chemistry analyzer (Beckman AU680, Brea, CA, USA).

#### 2.5.3. Histological Examination

Following animal dissection, liver, spleen, kidney, and testis specimens were harvested for histological analysis. Organs were fixed in 4% paraformaldehyde general-purpose fixative for 24 h. Then organs were sequentially dehydrated and degreased using standard histological methods, embedded in paraffin wax, and cut into continuous paraffin sections (4 μm) using a microtome, and the sections were stained with hematoxylin and eosin (HE), then examined under a microscope (Beyotime Biotechnology Co., Ltd., Shanghai, China).

### 2.6. Secondary Metabolomics Analysis

After 50 mg of BAAQ and UAAQ powders were weighed, 1.2 mL of 70% methanol pre-cooled to −20 °C containing 1 mg/L 2-chlorophenylalanine as an internal standard was added to the mixture with vortex for 30 s once every 30 min, a total of 6 times. The mixture was centrifuged at 12,000× *g* and 4 °C for 10 min, then the supernatant was collected and filtered through 0.22 μm filter for use. A UPLC-ESI-MS/MS system equipped with an ExionLC™ AD ultra-performance liquid chromatography unit (SCIEX, Framingham, MA, USA) and an Applied Biosystems 4500 Q TRAP mass spectrometer (SCIEX, Framingham, MA, USA) was employed for the analysis of secondary metabolites. Chromatographic separation was carried out using an Agilent SB-C18 column (1.8 µm, 2.1 mm × 100 mm; Agilent Technologies, Santa Clara, CA, USA). R software (version 4.2.0; R Foundation for Statistical Computing, Vienna, Austria) was used for data analysis.

### 2.7. 16S rRNA Sequencing

Fresh caecal contents of rats were collected in a sterile environment. After rapid freezing in liquid nitrogen, a total of 42 caecal samples in six groups were stored at −80 °C until being used. Using the bacterial genomic DNA extraction kit, the total genome DNA of bacteria was extracted and purified from the caecal contents. The 16S rRNA (V3-V4 regions) were amplified using the following specific primers (314F [5′-CCTAYGGGRBGCASCAG-3′] and 806R [5′-GGACTACNNGGGTATCTAAT-3′]) with the barcode. The amplicons were obtained by PCR, and the concentration and integrity of amplicons were detected using 2% agarose gel electrophoresis and purified with universal DNA kit. According to the characteristics of the amplified 16S region, the sequencing library was constructed using the TruSeq^®^ DNA PCR-Free Sample Preparation Kit (Illumina, San Diego, CA, USA) and evaluated by Qubit^®^ 2.0 Fluorometer (Thermo Scientific, Waltham, MA, USA) and Illumina NovaSeq6000 (Illumina, San Diego, CA, USA) [37].

Raw sequences were filtered to obtain high quality reads using Fastp software (V0.22.0, https://github.com/OpenGene/fastp, accessed on 9 September 2025), and constructed to get clean data using FLASH software (V1.2.11, http://ccb.jhu.edu/software/FLASH/, accessed on 9 September 2025). Following the detection of chimeras, the Uparse algorithm (USEARCH software V7, http://www.drive5.com/uparse/, accessed on 9 September 2025) consolidated the remaining high-quality sequences into operational taxonomic units (OTUs) exhibiting 97% sequence identity. To analyze the sequence data and create the histogram and heat map, QIIME 2 (version 2022.2; https://qiime2.org, accessed on 9 September 2025) and R software were used. Alpha-diversity (Chao1, Shannon and PD_whole_tree) and beta-diversity including principal coordinate analysis (PCoA), principal component analysis (PCA) and unweighted pair-group method with arithmetic means (UPGMA) clustering analysis using R software was used to reveal differences in species composition and community structure among samples. Linear discriminant analysis (LDA) effect size (LEfSe) tool was used to statistically evaluate group differences.

### 2.8. Statistical Analysis

All determinations were repeated three times. Data were expressed as mean ± standard deviation (SD). One-way ANOVA, Kruskal–Wallis H test and t-test were used for the statistical analysis using SPSS 27.0 (SPSS Inc., Chicago, IL, USA). GraphPad Prism (version 8.0, GraphPad Software, San Diego, CA, USA) was used to draw the graphs. *p* < 0.05 indicated statistical significance.

## 3. Results

### 3.1. The Effect of Boiling Treatment on the Quality Evaluation of A. argyi

#### 3.1.1. Sensory Evaluation and Chlorophyll Content

As shown in Table 1 and Table 2, boiling treatment significantly changed the color and flavor characteristics of *A. argyi* fresh leaves, frozen leaves and their BAAQ powders compared with that of the control UAAQ powder. The BAAQ powder derived from unfrozen fresh leaves surpassed that made from frozen leaves in aspects such as leaf shape and texture, as well as color and flavor. Compared with that of the control UAAQ group, the bitterness and flavor slightly decreased after alkaline boiling. Visual score combined with chlorophyll quantitative analysis revealed that boiling in alkaline NaHCO_3_ solution effectively maintained the green color of *A. argyi* leaves. The thermal stability of chlorophyll a is poorer than that of chlorophyll b, and the processing time needs to be controlled to avoid excessive loss of nutrients and active ingredients.

#### 3.1.2. Detection of Active Ingredients and Pharmacological Substances

The contents of flavonoids, polyphenols, volatile oil and its representative pharmacological components eucalyptol, borneol, camphor and α-thujone in *A. argyi* leaf powders were analyzed and shown in Table 3. The flavonoids, polyphenols and volatile oil of control (UAAQ powder) were significantly higher than these BAAQ powders (*p* < 0.05), and the eucalyptol, borneol, camphor and α-thujone contents were 0.031‰, 0.0691‰, 0.018‰ and 0.093‰, respectively. The data revealed that alkaline thermal processing statistically reduced these bioactive constituents in BAAQ powders, with water-soluble polyphenols exhibiting the most pronounced reduction (*p* < 0.05). When the alkali boiling time reached or exceeded 2 min, the presence of eucalyptol, borneol, camphor and α-thujone in the volatile oil of BAAQ powder was undetectable. Based on the above results, an alkaline boiling time of 2 min was selected as the most ideal time.

### 3.2. The Effect of Drying Treatment on the Quality Evaluation of A. argyi

Table 4 presents the outcomes regarding the sensory evaluation, chlorophyll, active and pharmacological components of *A. argyi* leaf powders under different drying conditions. Compared to the control UAAQ powder, BAAQ powders exhibited significantly higher chlorophyll content following hot air-drying, while demonstrating lower levels of flavonoids, polyphenols and volatile oils than UAAQ under both hot air-drying and freeze-drying (*p* < 0.05). The increase in drying temperature initially elevated the levels of chlorophyll, flavonoids, polyphenols and volatile oil in BAAQ powder, followed by a subsequent decline. It is worth noting that freeze-drying could retain more active components. This indicated that the hot air-drying at 40 °C and freeze-drying might be conducive to the retention and enrichment of these bioactive and pharmacological components in *A. argyi* leaves, providing valuable insights for the optimization of drying processes for the utilization of *A. argyi* for potential food or pharmaceutical applications. The BAAQ powder, prepared by alkaline boiling for 2 min followed by freeze-drying treatment, and UAAQ powder were used for the next nutritional composition analysis, acute oral toxicity test and secondary metabolomics analysis.

### 3.3. The Nutritional Value for Edible Raw Materials of A. argyi

Table S2 presents the evaluation of the nutrient composition of the BAAQ powder. The results showed that the tender leaves of *A. argyi* after boiling treatment had high crude protein (28.57 g/100 g), crude fat (11.26 g/100 g) and crude fiber (23.12 g/100 g), and contained β-carotene (2.93 mg/100 g), vitamin C (7.99 mg/100 g) and vitamin B_2_ (1.95 mg/100 g). Additionally, it was rich in minerals including calcium (1.71 g/100 g), sodium (841 mg/100 g), potassium (305 mg/100 g), manganese (44.4 mg/100 g), iron (8.81 mg/100 g), zinc (6.01 mg/100 g), aluminum (3.56 mg/100 g), selenium (3.5 µg/100 g) and copper (1.83 mg/100 g).

### 3.4. The Acute Toxicity of Edible Raw Materials of A. argyi in Rats

#### 3.4.1. Effects on Body Weight and Organ Index

**Figure 2:**
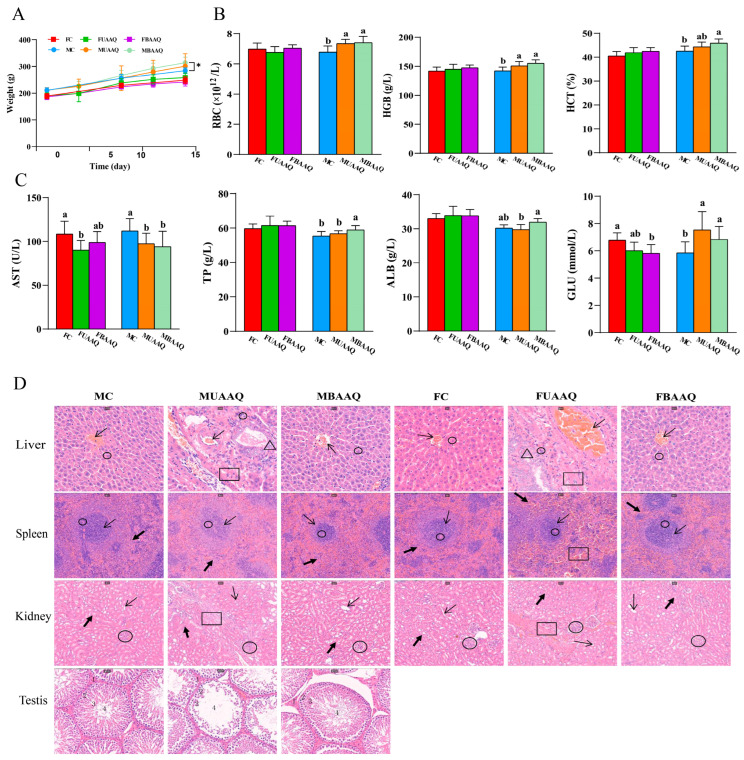
Body weight, blood indexes and organ histological changes of rats. (**A**) Body weight trend of rats. (**B**) Hematological indexes of rats with significant changes. (**C**) Serum biochemical indexes of rats with significant changes. (**D**) Histological sections of liver, spleen, kidney and testis in rats on day 14 (*n* = 5). Liver (300×), thin arrow shows central vein, triangle shows vacuoles, circle shows hepatocytes, square shows fibrosis, and the space between hepatocytes is the hepatic sinusoid. Spleen (100×), thin arrow shows white pulp, thick arrow shows red pulp, circle shows splenic body, square shows dilated splenic sinusoid. Kidney (100×), thin arrow shows distal tubule, thick arrow shows proximal tubule, circle shows renal corpuscle, square shows fibrosis, and the space between hepatocytes is the hepatic sinusoid. Testis (200×), 1: testicular interstitium, 2: epithelial cells, 3: spermatocyte of various levels, 4: sperm. FC, female control group; FUAAQ, female untreated *A. argyi* powder group; FBAAQ, female boiled *A. argyi* powder group; MC, male control group; MUAAQ, male untreated *A. argyi* powder group; MBAAQ, male boiled *A. argyi* powder group. Asterisks represent significant differences; different lowercase letters represent significant differences.

Figure 2A illustrated the body weight of rats treated without or with the indicated AAQ. With the progression of experiment, the body weight of rats all increased during the 14-day observation period. The values of body weight of male rats were higher than that of female rats. As shown in Table 5, the body weight of female rats was not significantly different among the FC, FUAAQ and FBAAQ group. The body weight of male rats in the MBAAQ group on day 14 (313.94 ± 20.71 g) was considerably greater than that of the MC group (*p* < 0.05). It is worth noting that three rats in the FUAAQ group died within one week after the oral administration, and one rat experienced difficulty to breath, but recovered later. In addition, there was one rat in the MUAAQ group died within one week, and four rats developed symptoms such as dyspnea, bloating, and diarrhea, of which three recovered and one had sustained diarrhea and weight loss until the end of the experiment period. No mortality or adverse symptoms were observed in boiled leaf treatment groups FBAAQ, MBAAQ and control group.

The body weight, food intake and organ index of rats on day 14 are shown in Table S3. The MBAAQ group exhibited a significantly greater weight gain compared to the MC and MUAAQ group (*p* < 0.05), while female groups showed no significant differences. The organ weights of the liver, spleen, kidney, testis or epididymis were not different among the groups, suggesting that there were no treatment-related changes.

#### 3.4.2. Effects on Hematology

The results of hematology analyses of rats are shown in Table S4 and Figure 2B. In male rats, MUAAQ and MBAAQ treatments significantly increased RBC and HGB levels compared with the control group (*p* < 0.05). No significant alterations occurred in PLT, MPV, PCT%, MCV, MCH, MCHC, NEUT%, LYMPH%, MONO%, EO% or BASO% levels among treatment groups.

#### 3.4.3. Effects on Serum Biochemical Indexes

The serum biochemical parameters of rats were demonstrated in Table S5 and Figure 2C. In female rats, FUAAQ treatment significantly decreased AST levels in comparison to FC, while FBAAQ treatment decreased GLU level. In males, MBAAQ treatment significantly increased TP and GLU levels, while AST level of rats in the MBAAQ group decreased compared to that in MC group (*p* < 0.05). Rats in the MUAAQ group exhibited lower AST and higher GLU levels than that in the MC group (*p* < 0.05), and higher TP and ALB levels than that in the MUAAQ group (*p* < 0.05). The ALT, ALP, GGT, BUN, CREA, CHOL, TC, HDL-C or LDL-C levels were not different among these treatment groups.

#### 3.4.4. Histopathological Examination

The HE staining of liver, spleen, kidney, testis tissue sections of rats treated with BAAQ and UAAQ were examined as shown in Figure 2D. Inflammatory cell infiltration and histological changes were observed in the liver, spleen, kidney and testicular tissues of the UAAQ group. Compared with that in the control group, liver tissue sections of UAAQ group showed obvious dislocation of hepatic cords, irregular cell arrangement, morphological changes, interstitial fibrosis, vacuolar degeneration and contraction of hepatic nuclei. There were no obvious morphological changes in BAAQ group. In comparison to that in control group, the spleen in the UAAQ group exhibited injury characterized by the presence of splenic cleats, an indistinct boundary between red and white pulp, dilation of spleen blood sinuses, and a reduction in both the number and volume of white pulp cells. The boundary between red pulp and white pulp was obvious in BAAQ and control groups, and no significant morphological changes were observed. Compared with the sections obtained from the control group, the sections of the renal cortex and medulla in the UAAQ group showed mild vascular degeneration, slight proliferation of glomerular capillaries, vacuolation of distal tubules, loss of proximal tubule structure, and fibrosis. There were no obvious morphological changes in the BAAQ group. In comparison to the sections from the control group, the spermatogenic tubules in the BAAQ group were orderly, with an intact basic membrane of the spermatogenic epithelium. The spermatogenic cells at all levels were neatly organized, and interstitial congestion was not observed. The spermatogenic cells were basically arranged normally in the basal membrane. In the UAAQ group, the morphological structure of the spermatogenic tubules underwent some changes. The basal membrane was incomplete. The number of spermatogenic cell layers decreased, the cells became sparse and there were few interstitial cells, which were congested. However, the arrangement of spermatogenic cells within the basal membrane was basically normal.

### 3.5. Secondary Metabolites Profiles in Fresh Tender Leaves of A. argyi

**Figure 3:**
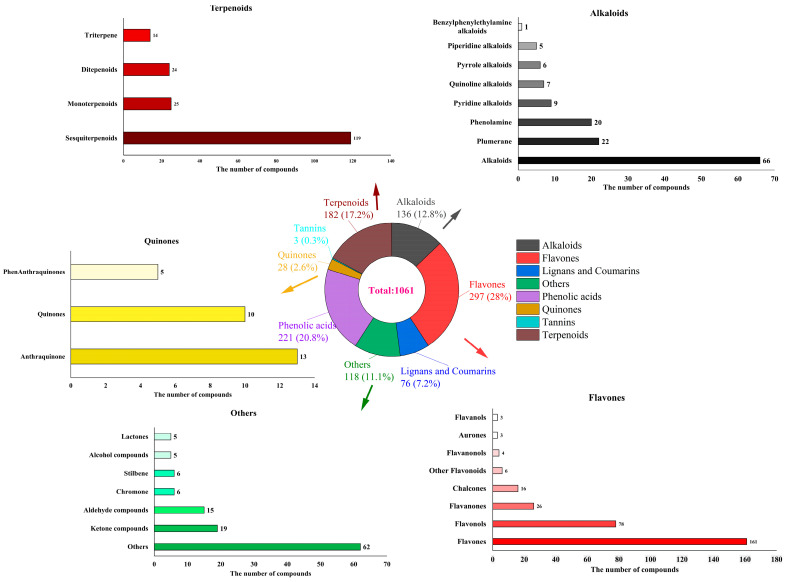
Ring graph of metabolite class composition of tender leaves of *A. argyi*.

**Figure 4:**
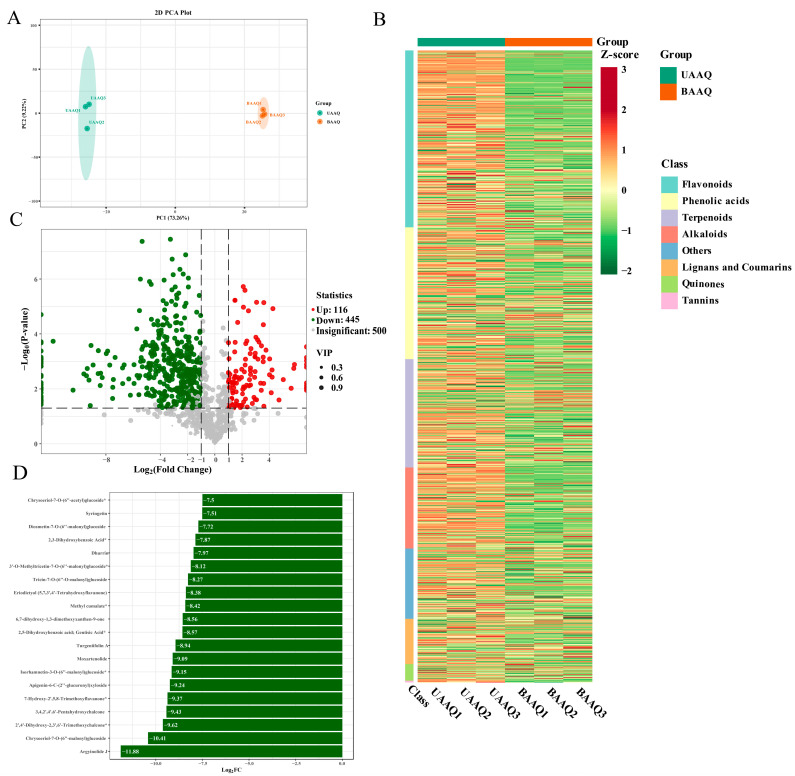
Secondary metabolite profiles analysis of untreated *A. argyi* (UAAQ) powder and boiled *A. argyi* (BAAQ) powder. PCA score plot (**A**). Overall heat map (**B**). Volcanic map (**C**). Differential metabolite bar chart of top 20 (**D**). PCA, principal component analysis.

Based on the UPLC-ESI-MS/MS detection platform, the MRM mode of triple quadrupole mass spectrometry and the MWDB database were used to analyze the secondary metabolites profiles in the fresh and tender leaves of *A. argyi*. As shown in Figure 3, a total of 1061 metabolites were identified, including 297 flavonoids, 221 phenolic acids, 182 terpenoids, 136 alkaloids, 76 lignans and coumarins, 28 quinones, 3 tannins, and 118 others metabolites. The different metabolites between UAAQ and BAAQ were compared as shown in Figure 4A. Six principal components were obtained, among which the contribution rates of PC1 and PC2 were 64.68% and 10.01%, respectively. Notably, metabolites in UAAQ and BAAQ samples were clustered differently, clearly indicating a trend of metabolomic separation between UAAQ and BAAQ. As shown in Figure 4B, the differential metabolites of UAAQ were mainly found in the red high-expression area, whereas those of BAAQ were mainly located in the green low-expression area. This demonstrates significant differences in the metabolite profiles of UAAQ and BAAQ. Based on the results of OPLS-DA, metabolites with VIP > 1 and *p* < 0.05 were selected as significant differential metabolites, and the volcano map of the differential metabolites was generated as shown in Figure 4C. The comparison between UAAQ and BAAQ identified 561 differential metabolites, among which 116 metabolites were upregulated (20.7%) and 445 metabolites were downregulated (79.3%). Figure 4D shows the contents of metabolites in the top 20 of the difference multiples were down-regulated.

After alkaline boiling treatment, the contents of most secondary metabolites in tender leaves of *A. argyi* showed a decreasing trend. As shown in Table 6, 15 potentially harmful or irritating components, including 3-hydroxy-2-aminobenzoic acid (top 10 metabolites), dhurrin (top 20 metabolites), 3-amino-2-naphthoic acid, 2-nitrophenol, histamine, benzamide, isoalantolactone, n-benzylformamide, 3,4-dihydrocoumarin, genipin and turgeniifolin A (outside the top 20 metabolites) were significantly downregulated (*p* < 0.01). Among them, phthalic acid, 3-hydroxy-2-aminobenzoic acid and 3,4-dihydroxybenzoic acid were undetected in BAAQ powder after alkaline boiling, suggesting that the high-temperature alkaline treatment may significantly reduce these components through volatilization, degradation or structural transformation. Notably, that 3-O-methylgallic acid (an anthocyanin metabolite), which has antioxidant and anticancer activities, was significantly upregulated after alkaline boiling (*p* < 0.01).

### 3.6. Edible Raw Materials of A. argyi Modulated Caecal Microbiota Community in Rats

#### 3.6.1. Community Composition of the Caecal Microbiota in Rats

**Figure 5:**
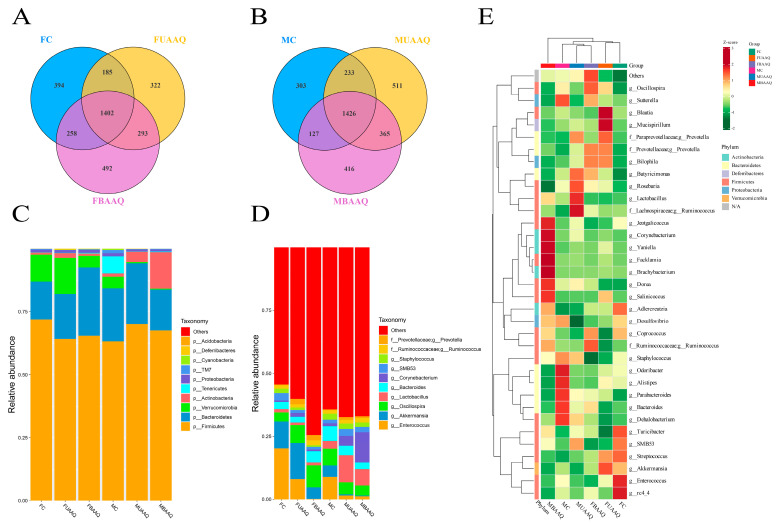
*A. argyi* treatments altered caecal microbiota structure in rats on day 14 (*n* = 7). Venn diagram illustrates overlaps among FC, FUAAQ and FBAAQ (**A**), and among MC, MUAAQ and MBAAQ (**B**). Bacteria composition of different communities at phylum level (**C**) and genus level (**D**). Community heat map at genus level (**E**). FC, female control group; FUAAQ, female untreated *A. argyi* powder group; FBAAQ, female boiled *A. argyi* powder group; MC, male control group; MUAAQ, male untreated *A. argyi* powder group; MBAAQ, male boiled *A. argyi* powder group.

The V3-V4 region of the 16S rRNA gene was sequenced to characterize the caecal microbiota of rats in the FC, FUAAQ, FBAAQ, MC, MUAAQ and MBAAQ groups at day 14. According to the Venn diagram Figure 5A,B, a total of 27,553 and 28,992 OTUs were identified in the caecal contents of female and male rats, respectively. Specifically, 948 common OTUs were identified across the three treatments in female rats, whereas 7937 unique OTUs were found in the control group, 7316 in the FUAAQ group and 10,712 in the FBAAQ group. In male rats, the corresponding numbers were 924, while the control, MUAAQ, and MBAAQ groups exhibited 6114, 10,879, and 10,445 unique OTUs, respectively. The results demonstrated that BAAQ treatment in female rats, as well as both BAAQ and UAAQ treatment in male rats, increased the number of OTUs in the caecal microbiota.

The composition of the caecal microbiota at both the phylum and genus levels exhibited significant alterations due to the treatment of *A. argyi* (Figure 5C,D). At the phylum level, the dominant microbiota consisted of Firmicutes, Bacteroidota, Verrucomicrobiota, Actinobacteriota, Tenericutes, Proteobacteria, TM7, Cyanobacteria, Deferribacteres, and Acidobacteres (Figure 5C). In female rats, treatment with BAAQ and UAAQ increased the abundance of phylums Bacteroidota and Actinobacteriota, and decreased that of Firmicutes and Verrucomicrobiota, as well as the Firmicutes/Bacteroidetes (F/B) ratio (*p* < 0.05). In male rats, BAAQ and UAAQ treatment significantly decreased the abundance of Verrucomicrobiota and Tenericutes, increased the abundance of Firmicutes and Actinobacteriota and elevated the F/B ratio compared to the MC group (*p* < 0.05). At the genus level, BAAQ and UAAQ treatments significantly increased the abundance of *Oscillospira* and *Bacteroides* in female rats and *Corynebacterium* and *Lactobacillus* in male rats when compared to the controls (*p* < 0.05). BAAQ and UAAQ treatments significantly reduced the abundances of *Enterococcus*, *Akkermansia* and *Staphylococcus* in female rats (*p* < 0.05), whereas only *Enterococcus* and *Akkermansia* were significantly decreased in male rats (*p* < 0.05) (Figure 5D). The abundances of *Enterococcus* and *Staphylococcus* were significantly decreased in MBAAQ treatments compared with that of the control group, while *Lactobacillus* was significantly increased (*p* < 0.05) (Figure 5E).

#### 3.6.2. Microbial Diversity of the Caecal Contents in Rats

**Figure 6:**
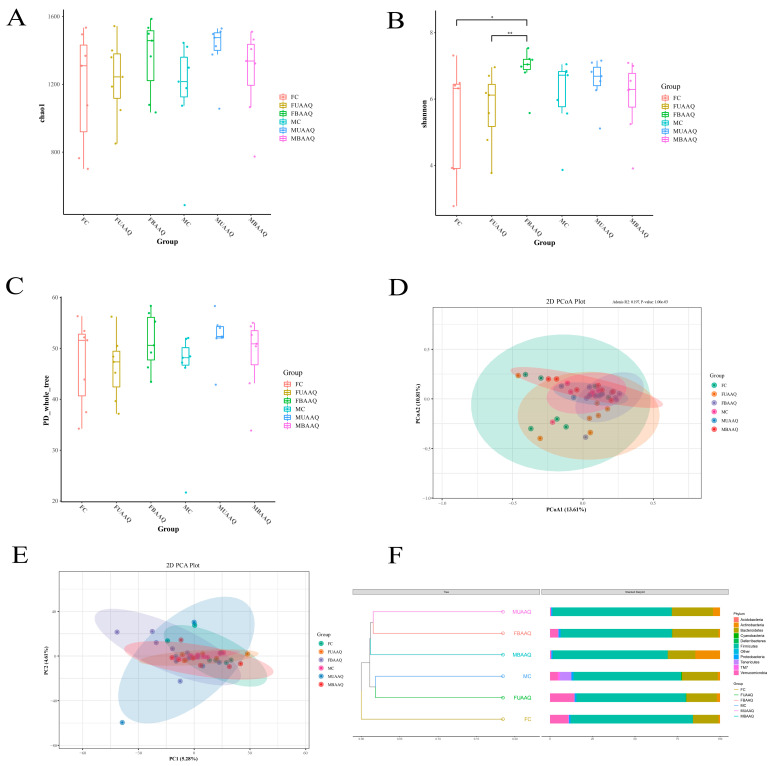
Effects of *A. argyi* treatment on α- and β-diversity of caecal microbiota in rats on day 14 (*n* = 7). α-diversity of caecal microbiota includes chao1 (**A**), Shannon (**B**) and PD_whole_tree (**C**) among the six groups. β-diversity includes PCoA (**D**), PCA (**E**) and UPGMA. Clustering was conducted based on Unweighted Unifrac distance (**F**). PD, phylogenetic diversity; PCoA, principal coordinate analyses; PCA, principal component analysis; UPGMA, unweighted pair-group method with arithmetic means. FC, female control group; FUAAQ, female untreated *A. argyi* powder group; FBAAQ, female boiled *A. argyi* powder group; MC, male control group; MUAAQ, male untreated *A. argyi* powder group; MBAAQ, male boiled *A. argyi* powder group.

The treatments of fresh and tender leaves of *A. argyi* altered the α- and β-diversity of caecal microbiota community in rats on day 14. α-diversity included Chao1, Shannon and PD_whole_tree, as shown in Figure 6A–C. Shannon in FBAAQ group was significantly higher than that in FC and FUAAQ groups (*p* < 0.05), suggesting enhanced species richness and phylogenetic diversity (Figure 6B). β-diversity analysis by PCoA, PCA and UPGMA further highlighted structural differences in microbial communities (Figure 6D–F). The PCA data variations among samples were small as shown in Figure 6E, which requires to combine with PCoA to make a comprehensive judgment. PCoA based on Bray–Curtis distances demonstrated clear separation between AAQ-treated groups and control group (PERMANOVA, *p* < 0.001), indicating that there were statistically significant differences in the microbial community structures among different groups (Figure 6D). UPGMA clustering tree results showed that there were significant differences in cecal bacterial community among female rats (Figure 6F, *p* < 0.001).

#### 3.6.3. Analysis of Differential Caecal Microbiota in Rats

**Figure 7:**
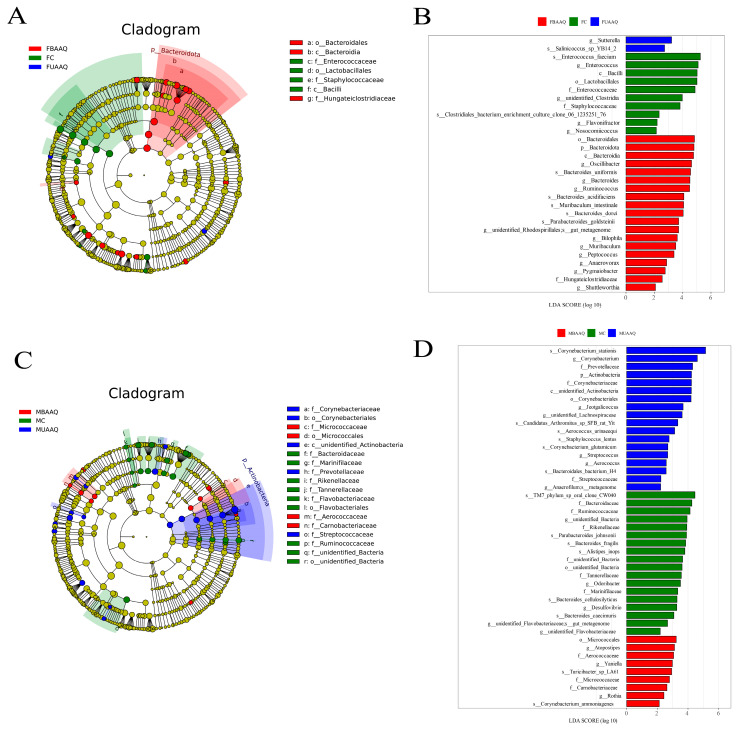
*A. argyi* treatments caused significant alterations in caecal microbiota in rats on day 14 (*n* = 7). Cladogram obtained by LEfSe analysis displaying species of caecal microbiota that can be distinguished among female rats (**A**) and male rats (**C**) with LDA scores > 2 (**B**,**D**). LDA, linear discriminant analysis; LEfSe, LDA effect size; FC, female control group; FUAAQ, female untreated *A. argyi* powder group; FBAAQ, female boiled *A. argyi* powder group; MC, male control group; MUAAQ, male untreated *A. argyi* powder group; MBAAQ, male boiled *A. argyi* powder group.

The LEfSe was utilized to identify the caecal microbiota taxa with significant differences of relative abundance in rats treated with different *A. argyi* preparations (Figure 7). Rats treated with AAQ powders showed twenty-one (Figure 7A,B) and twenty-seven (Figure 7C,D) unique bacteria in female and male rats, respectively. In female rats, the phylum Bacteroidota, the genus *Ruminococcus*, *Bacteroides* and *Oscillibacter* in the FBAAQ group were the differential bacteria, while those in FC were in the genus *Enterococcus* and *Clostridia* (Figure 7A,B, *p* < 0.05). In male rats, the phylum Actinobacteria and the genus *Corynebacterium*, the genera *Atopostipes* and *Yaniella*, and the genera *Bacteroides*, *Odoribacter* and *Desulfovibrio* were dominant in the MUAAQ, MBAAQ and MC groups (Figure 7C,D, *p* < 0.05), respectively.

**Figure 8:**
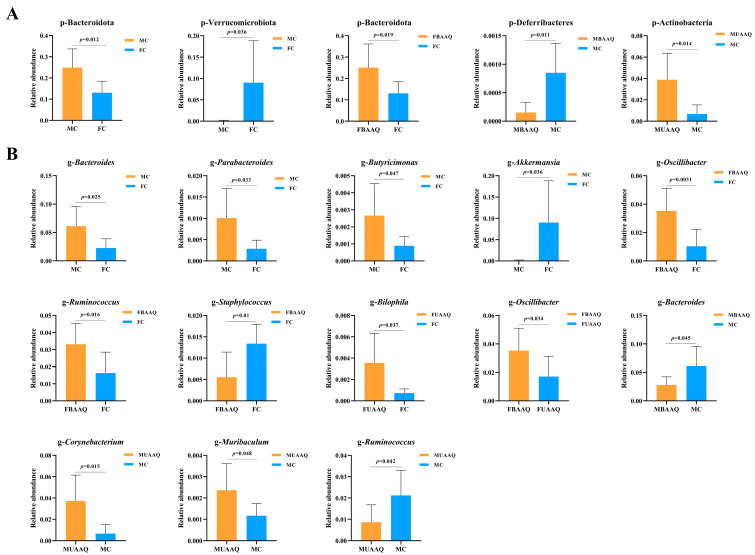
Significant changes of differential caecal microbiota at phylum (**A**) and genus level (**B**) in rats on day 14 (*n* = 7). FC, female control group; FUAAQ, female untreated *A. argyi* powder group; FBAAQ, female boiled *A. argyi* powder group; MC, male control group; MUAAQ, male untreated *A. argyi* powder group; MBAAQ, male boiled *A. argyi* powder group.

The significant changes of differential caecal microbiota at phylum level in rats are shown in Figure 8A. The caecal microbiota of male and female rats in the control group exhibited significantly elevated levels of Bacteroidota (*p* = 0.012) and Verrucomicrobiota (*p* = 0.036), respectively. BAAQ treatment significantly increased the relative abundances of Bacteroidota (*p* = 0.019) in female rats, while decreased the relative abundances of Deferribacteres (*p* = 0.011) in male rats. Moreover, compared to that in the MC group, the Actinobacteria in the MUAAQ group exhibited a substantial increase (*p* = 0.014). Figure 8B illustrates the considerable alterations in the caecal microbiota at the genus level in rats. The caecal microbiota of male rats in the control group exhibited significantly higher relative abundances of *Bacteroides* (*p* = 0.025), *Parabacteroides* (*p* = 0.033), and *Butyricimonas* (*p* = 0.047), alongside a significant reduction in *Akkermansia* (*p* = 0.036) as compared to that in the female controls. In female rats, compared with that in FC, UAAQ treatment increased the relative abundance of *Bilophila* (*p* = 0.037). In contrast, BAAQ treatment in female rats markedly elevated the relative abundance of *Oscillibacter* (*p* = 0.0031) and *Ruminococcus* (*p* = 0.016), while reducing *Staphylococcus* (*p* = 0.01) in comparison to FC. Furthermore, BAAQ treatment enhanced *Oscillibacter* (*p* = 0.0033) relative to the UAAQ. In male rats, UAAQ treatment, in comparison to the control group, elevated the relative abundance of *Corynebacterium* (*p* = 0.015) and *Muribaculum* (*p* = 0.048), while diminishing the *Ruminococcus* (*p* = 0.042). BAAQ treatment decreased the relative abundance of *Bacteroides* (*p* = 0.045) in male rats. These differences reflect the effects of tender leaves of *A. argyi* treatment and gender on the relative abundance of specific microbiota.

## 4. Discussion

*A. argyi* has significant health benefits and culinary attributes, making it a potential choice for future food sources. Therefore, it is essential to develop a safe method for the preparation of *A. argyi* for food usage, necessitating a thorough examination. According to the published books and research articles, field visits to local inhabitants and the optimization outcomes of this study, the edible portion of *A. argyi* include the tender stems, buds and leaves harvested during the seedling and growth stages. These materials are treated with NaHCO_3_ in boiling water for 2–5 min, then soaked in cold water for 30 min in preparation for associated culinary products. If these materials are intended for prolonged use, after soaking, the tender leaves need to be dried at low temperature or crushed and stored. Alternatively, after soaking, the tender leaves can be cut into small pieces, then broken them into vegetable puree and stored at low temperature. In this process, alkaline substances may neutralize organic acids in the tender leaves, protect chlorophyll from degradation and thereby maintain the vibrant green color. Alkaline boiling and soaking reduce the concentration of volatile oils and certain bioactive components, hence reducing bitterness. Furthermore, this treatment also softens the texture of the tender leaves, making them more malleable and appropriate for following processing stages like crushing or homogenization.

The sensory evaluation and flavor analysis of *A. argyi* are crucial for its further development [38]. In order to ensure the safety of tender leaves of *A. argyi* as a food raw material, the nutritional and active components were determined and toxicological assessment was carried out. The results showed that the tender leaves of *A. argyi* are rich in protein, dietary fiber, vitamins and minerals. The changing trend of active components during the alkali boiling treatment and drying process indicated that heat treatment caused a substantial reduction in bioactive components (*p* < 0.05), with the loss of water-soluble polyphenols being the most pronounced. Under alkaline boiling conditions, the volatile oil content decreased in a dose-dependent manner. It was worth noting that after alkaline boiling treatment for more than 2 min, both α-thujone and camphor (constituents of volatile oil) fell below the detection limit. Previous studies have shown that α-thujone and camphor may have neurotoxicity at high doses, primarily via the inhibition of γ-aminobutyric acid receptors and the induction of oxidative stress [39]. When tender leaves of *A. argyi* were boiled in an alkaline solution, most of the volatile oil might be volatilized or evaporated into the air with steam, or dissolved in water. This suggests that alkaline boiling for over 2 min may produce safe and edible *A. argyi* powder.

Toxicological evaluation confirmed the safety of *A. argyi* tender leaves after the alkaline boiling treatment as food raw materials, with no adverse effects. The BAAQ powder was prepared mainly by alkaline boiling for 2 min, followed by freeze-drying, whereas the UAAQ powder was produced mainly via freeze-drying treatment. The acute toxicity assessment indicated that no mortality or adverse effects were observed in both male and female rats received a gavage dosage of 7.5 g/kg·BW BAAQ powder, but three rats in the FUAAQ group and one rat in the MUAAQ group died within one week. According to the analysis of secondary metabolites, alkaline boiling treatment not only reduced volatile oils, α-thujone and camphor in tender leaves of *A. argyi*, but also considerably downregulated potentially hazardous or irritating components. 3-O-methylgallic acid was increased in BAAQ powder may be attributed to the chemical reactions such as hydrolysis and isomerization triggered by the alkaline boiling process, which promoted the transformation of precursor substances into this active component.

The hematological and biochemical parameters are crucial for evaluating the physiological and pathological conditions of animals. Despite the UAAQ group of rats exhibiting clinical indications of mortality and diarrhea, their hematological and biochemical characteristics did not substantially vary from those of the BAAQ group on day 14. Only the rats in the MBAAQ group had elevated levels of TP and ALB in comparison to that in the MUAAQ group (*p* < 0.05). TP is composed of ALB and globulin and elevated levels of TP and ALB usually indicate a good nutritional state [40]. This is consistent with the result that rats in the MBAAQ group had a significantly greater weight gain in comparison to that in the MC and MUAAQ groups. Furthermore, other hematological and biochemical markers, such as RBC, HGB, HCT, AST and GLU, were within their normal ranges according to internationally accepted Standards for Veterinary Clinical Pathology guidelines [41], indicating the lack of abnormalities in these animals. Therefore, the BAAQ powder was categorized as practically non-toxic (Grade 1) according to the Chinese National Food Safety Standard for acute oral toxicity test. However, the safety of long-term consumption of *A. argyi* tender leaves needs to be further verified.

The potential effects of changes in intestinal microenvironment are closely related to intestinal injury [42]. The structural differences of microorganisms alter their functions, which are closely related to the health of the host [43]. According to previous studies, phenolic acids and flavonoids were the main active substances in *A. argyi*, which have significant anti-inflammatory, antioxidant and antibacterial effects, and play positive roles in the regulation of gut microbiota [44,45,46,47,48]. The contents of *A. argyi* leaves could play a positive role by regulating the F/B ratio, as well as increasing the abundance of Verrucomicrobiota [48]. This was consistent with the results of microbial abundance at phylum level in our study. The F/B ratio was significantly increased in male rats and decreased in female rats treated with *A. argyi* compared with that in the control rats. A moderate increase in the F/B ratio can enable the intestinal microbiota to obtain energy more effectively and promote the synthesis of fat and cholesterol. BAAQ treatment showed profound effects on microbial composition, markedly reducing the abundance of potentially harmful bacteria, such as *Enterococcus* and *Staphylococcus*, while increasing the abundance of the beneficial *Lactobacillus*, *Oscillibacter* and *Ruminococcus*. *Enterococcus* is an important opportunistic pathogen that can cause various infections [49,50]. *Lactobacillus* is commonly found in the gastrointestinal tracts of humans and animals and is crucial for maintaining the health of the host [51,52,53]. *Lactobacillus* supports intestinal health by generating a series of short-chain fatty acids (SCFAs) [54]. SCFAs, especially butyrates, have beneficial effects on regulating intestinal immune function and inhibiting intestinal inflammation [55,56]. In addition, the increased abundance of *Ruminococcus* can increase the concentration of SCFAs, thereby protecting the intestinal barrier function and exerting anti-inflammatory properties in the host [55]. The treatment of UAAQ increased the abundance of *Bilophila*, *Corynebacterium* and *Muribaculum*, and decreased the beneficial bacteria *Ruminococcus*. The Gram-positive, GC-rich *Corynebacterium* genus is a common opportunistic pathogen that can cause opportunistic infections in hosts with weakened immune systems and may be resistant to multiple drugs. Overall, these findings suggest that BAAQ treatment might promote the proliferation of beneficial intestinal microbiota while inhibiting the growth of potentially pathogenic and harmful bacteria, thereby enhancing the production of beneficial metabolites such as SCFA. However, UAAQ may cause intestinal inflammation. Therefore, a safe and nutritious preparation process of *A. argyi* tender leaves is essential for its use in foods.

## 5. Conclusions

This study developed a safe method to prepare tender leaves of *A. argyi* planted in Hubei Province for food use. The fresh and tender leaves of *A. argyi* were treated with alkaline boiling, soaked in cold water, dried at low temperature and crushed into vegetable powder, or cut and broken into vegetable puree. It demonstrated that BAAQ powder exhibited practically non-toxic (LD_50_ > 7.5 g/kg BW). Alkaline boiling and soaking treatments not only effectively reduced volatile oils, α-thujone and camphor while enhancing the organoleptic properties of BAAQ powder, but also considerably downregulated potentially hazardous or irritating components. The administration of BAAQ powder to rats regulated the composition of the caecal microbiota by enhancing beneficial taxa (*Lactobacillus*, *Oscillibacter* and *Ruminococcus*) and suppressing opportunistic pathogens (*Enterococcus* and *Staphylococcus*), with sex-specific effects on microbial diversity and taxonomic characteristics. This study provides key data support for the future food application of *A. argyi* tender leaves and its industrialization potential is worth further exploration.

## Figures and Tables

**Table 1:** Sensory evaluation of fresh *A. argyi* leaves under different processing times.

Group	UAAQ	BAAQ				
	Control	Boiling 1 min	Boiling 2 min	Boiling 3 min	Boiling 4 min	Boiling 5 min
After-boiling	/	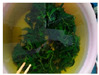	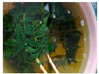	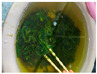	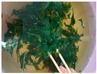	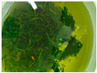
Soaked water	/	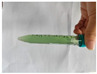	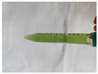	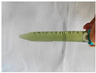	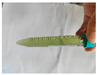	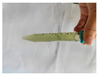
Before drying	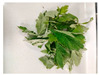	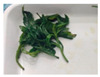	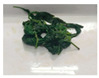	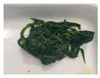	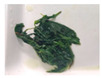	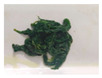
After drying	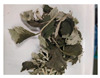	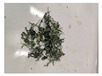	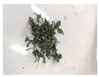	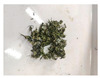	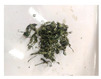	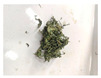
Powder	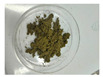	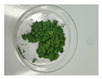	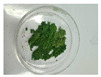	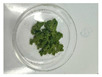	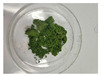	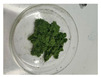
Chlorophyll extract	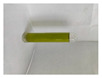	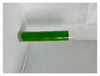	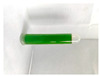	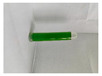	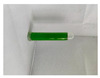	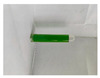
Chlorophyll a (mg/g)	1.78 ± 0.15 ^c^	2.28 ± 0.03 ^a^	2.26 ± 0.02 ^ab^	2.21 ± 0.04 ^ab^	2.14 ± 0.08 ^b^	2.22 ± 0.03 ^ab^
Chlorophyll b (mg/g)	0.62 ± 0.07 ^b^	2.03 ± 0.27 ^a^	2.01 ± 0.23 ^a^	1.88 ± 0.51 ^a^	2.46 ± 0.62 ^a^	2.08 ± 0.51 ^a^
Total Chlorophyll (mg/g)	2.40 ± 0.22 ^b^	4.31 ± 0.25 ^a^	4.27 ± 0.20 ^a^	4.09 ± 0.47 ^a^	4.61 ± 0.54 ^a^	4.30 ± 0.48 ^a^
Character description (powder)	Color: 2 Aroma intensity: 6 Bitterness intensity: 6 Bitterness persistence: 3	Color: 6 Aroma intensity: 7 Bitterness intensity: 5 Bitterness persistence: 3	Color: 6 Aroma intensity: 6 Bitterness intensity: 5 Bitterness persistence: 4	Color: 7 Aroma intensity: 6 Bitterness intensity: 4 Bitterness persistence: 5	Color: 7 Aroma intensity: 6 Bitterness intensity: 5 Bitterness persistence: 6	Color: 6 Aroma intensity: 6 Bitterness intensity: 5 Bitterness persistence: 6

Mean values with distinct letters in the same row showed significant different (*p* < 0.05). Data are expressed as means ± SD, *n* = 3. UAAQ and BAAQ powders were prepared by freeze-drying treatment. BAAQ, boiled tender leaves of *A. argyi* cv. Qiai; UAAQ, untreated tender leaves of *A. argyi* cv. Qiai.

**Table 2:** Sensory evaluation of frozen *A. argyi* leaves under different processing times.

Group	UAAQ	BAAQ				
	Control	Boiling 1 min	Boiling 2 min	Boiling 3 min	Boiling 4 min	Boiling 5 min
After-boiling	/	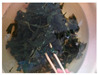	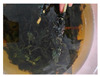	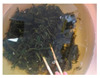	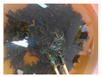	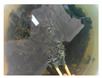
Soaked water	/	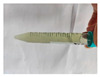	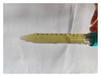	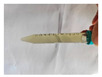	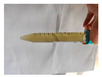	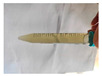
Before drying	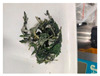	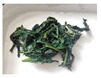	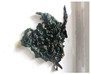	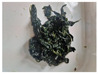	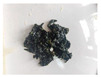	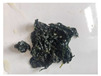
After drying	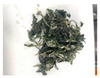	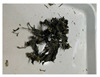	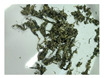	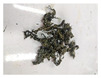	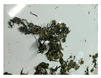	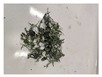
Powder	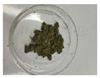	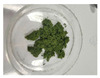	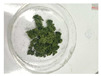	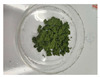	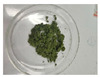	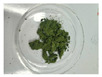
Chlorophyll extract	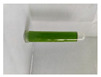	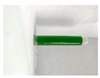	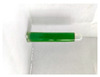	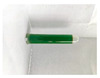	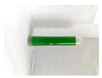	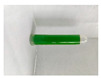
Chlorophyll a (mg/g)	2.15 ± 0.07	2.01 ± 0.07	2.15 ± 0.12	2.01 ± 0.10	2.11 ± 0.11	2.13 ± 0.13
Chlorophyll b (mg/g)	1.42 ± 0.21 ^b^	3.19 ± 0.31 ^a^	2.55 ± 0.57 ^a^	3.19 ± 0.34 ^a^	2.72 ± 0.44 ^a^	2.67 ± 0.59 ^a^
Total Chlorophyll (mg/g)	3.57 ± 0.28 ^b^	5.20 ± 0.24 ^a^	4.70 ± 0.45 ^a^	5.20 ± 0.25 ^a^	4.83 ± 0.33 ^a^	4.80 ± 0.46 ^a^
Character description (powder)	Color: 0 Aroma intensity: 8 Bitterness intensity: 7 Bitterness persistence: 7	Color: 6 Aroma intensity: 6 Bitterness intensity: 5 Bitterness persistence: 5	Color: 5 Aroma intensity: 6 Bitterness intensity: 5 Bitterness persistence: 6	Color: 7 Aroma intensity: 6 Bitterness intensity: 6 Bitterness persistence: 5	Color: 7 Aroma intensity: 6 Bitterness intensity: 5 Bitterness persistence: 6	Color: 6 Aroma intensity: 6 Bitterness intensity: 6 Bitterness persistence: 6

Mean values with distinct letters in the same row showed significant different (*p* < 0.05). Data are expressed as means ± SD, *n* = 3. UAAQ and BAAQ powders were prepared by freeze-drying treatment. BAAQ, boiled tender leaves of *A. argyi* cv. Qiai; UAAQ, untreated tender leaves of *A. argyi* cv. Qiai.

**Table 3:** Active and pharmacological components in AAQ powders under different boiling conditions.

Powder	Group	Flavonoids (mg/g)	Polyphenols (mg/g)	Volatile Oil (%)	Eucalyptol (‰)	Borneol (‰)	Camphor (‰)	α-Thujone (‰)
UAAQ	Control	13.04 ± 0.02 ^a^	11.59 ± 0.20 ^a^	0.230 ± 0.010 ^a^	0.031 ± 0.001	0.069 ± 0.001 ^a^	0.018 ± 0.001	0.093 ± 0.002
BAAQ	Boiling 1 min	11.01 ± 0.02 ^b^	4.73 ± 0.00 ^b^	0.173 ± 0.006 ^b^	-	0.015 ± 0.003 ^b^	0.012 ± 0.001	0.022 ± 0.002
	Boiling 2 min	9.21 ± 0.02 ^c^	3.49 ± 0.01 ^c^	0.107 ± 0.006 ^c^	-	-	-	-
	Boiling 3 min	7.23 ± 0.02 ^d^	3.04 ± 0.01 ^d^	0.100 ± 0.010 ^c^	-	-	-	-
	Boiling 4 min	5.72 ± 0.03 ^e^	2.93 ± 0.00 ^e^	0.083 ± 0.006 ^d^	-	-	-	-
	Boiling 5 min	4.92 ± 0.02 ^f^	2.66 ± 0.01 ^f^	0.063 ± 0.012 ^e^	-	-	-	-

Mean values with distinct letters in the same column showed significant different (*p* < 0.05). Data are expressed as means ± SD, *n* = 3. “-” indicated that the concentration in the corresponding group was lower than the detection limit of the detection method (eucalyptol 0.0006 mg/mL, borneol 0.0005 mg/mL, camphor 0.0002 mg/mL, α-thujone 0.0002 mg/mL). UAAQ and BAAQ powders were prepared by freeze-drying treatment. BAAQ, boiled tender leaves of *A. argyi* cv. Qiai; UAAQ, untreated tender leaves of *A. argyi* cv. Qiai.

**Table 4:** Chlorophyll, active and pharmacological components in AAQ powders under different drying conditions.

Group	25 °C Drying	40 °C Drying	65 °C Drying	Freeze-Drying	Control
Samples	BAAQ				UAAQ
	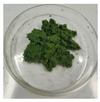	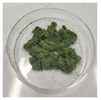	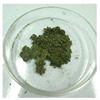	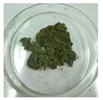	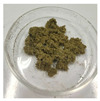
Character description (powder)	Color: 8 Aroma intensity: 5 Bitterness intensity: 6 Bitterness persistence: 5	Color: 7 Aroma intensity: 5 Bitterness intensity: 5 Bitterness persistence: 6	Color: 6 Aroma intensity: 4 Bitterness intensity: 4 Bitterness persistence: 5	Color: 3 Aroma intensity: 6 Bitterness intensity: 5 Bitterness persistence: 6	Color: 1 Aroma intensity: 8 Bitterness intensity: 8 Bitterness persistence: 7
Chlorophyll a (mg/g)	2.09 ± 0.11 ^a^	1.91 ± 0.16 ^ab^	1.75 ± 0.17 ^b^	1.11 ± 0.15 ^c^	1.30 ± 0.08 ^c^
Chlorophyll b (mg/g)	1.01 ± 0.14 ^a^	0.85 ± 0.12 ^ab^	0.79 ± 0.13 ^b^	0.51 ± 0.06 ^b^	0.74 ± 0.06 ^c^
Total Chlorophyll (mg/g)	3.10 ± 0.25 ^a^	2.76 ± 0.29 ^ab^	2.55 ± 0.30 ^b^	1.62 ± 0.21 ^c^	2.04 ± 0.14 ^c^
Flavonoids (mg/g)	13.10 ± 0.05 ^c^	15.05 ± 0.02 ^b^	12.77 ± 0.02 ^d^	13.04 ± 0.09 ^c^	19.00 ± 0.02 ^a^
Polyphenols (mg/g)	5.79 ± 0.00 ^d^	5.97 ± 0.00 ^c^	5.23 ± 0.25 ^e^	6.70 ± 0.00 ^b^	14.24 ± 0.00 ^a^
Volatile oil (%)	0.390 ± 0.026 ^c^	0.463 ± 0.021 ^b^	0.370 ± 0.010 ^c^	0.470 ± 0.010 ^b^	1.027 ± 0.006 ^a^
Eucalyptol (‰)	-	-	-	-	0.015 ± 0.000
Borneol (‰)	-	-	-	-	0.075 ± 0.002
Camphor (‰)	-	-	-	-	0.114 ± 0.002
α-Thujone (‰)	-	-	-	-	1.303 ± 0.021

Mean values with distinct letters in the same row showed significant different (*p* < 0.05). Data are expressed as means ± SD, *n* = 3. “-” indicated that the concentration in the corresponding group was lower than the detection limit of the detection method (eucalyptol 0.0006 mg/mL, borneol 0.0005 mg/mL, camphor 0.0002 mg/mL, α-thujone 0.0002 mg/mL). BAAQ powder was prepared by alkaline boiling for 2 min. BAAQ, boiled tender leaves of *A. argyi* cv. Qiai; UAAQ, untreated tender leaves of *A. argyi* cv. Qiai.

**Table 5:** Body weight and survival quantity of rats.

Groups		Body Weight (g)			Death/Treated Rats	Clinical Signs
		Day 1	Day 8	Day 14		
Female	FC	189.36 ± 9.34	229.03 ± 8.62 ^ab^	248.54 ± 12.62 ^ab^	0/10	None
	FUAAQ	190.41 ± 10.65	238.04 ± 16.23 ^a^	258.11 ± 17.48 ^a^	3/10	After the oral administration, 3 died within the first week, and 1 experienced difficulty to breath, but recovered.
	FBAAQ	186.18 ± 10.77	223.56 ± 8.15 ^b^	241.34 ± 15.04 ^b^	0/10	None
Male	MC	210.47 ± 9.85	255.92 ± 14.41	284.10 ± 11.02 ^b^	0/10	None
	MUAAQ	211.32 ± 9.77	256.18 ± 45.96	301.29 ± 46.02 ^ab^	1/10	After the oral administration, 1 died within the first week, and 4 showed symptoms of difficulty to breath, abdominal distension, and diarrhea. Three of 4 recovered, and the remaining one maintained the diarrhea and lost body weight until the end of the test.
	MBAAQ	211.29 ± 6.43	266.07 ± 19.02	313.94 ± 20.71 ^a^	0/10	None

Mean values with distinct letters in the same column showed significant different within the same gender (*p* < 0.05). Data are expressed as means ± SD, *n* = 10, except FUAAQ (*n* = 7) and MUAAQ (*n* = 9). FC, female control group; FUAAQ, female untreated *A. argyi* powder group; FBAAQ, female boiled *A. argyi* powder group; MC, male control group; MUAAQ, male untreated *A. argyi* powder group; MBAAQ, male boiled *A. argyi* powder group.

**Table 6:** Differential metabolites of untreated *A. argyi* powder and boiled *A. argyi* powder. (*n* = 3).

Systematic Name	Formula	Structure	Classification	BAAQ vs. UAAQ Type	Safety Risk
Phthalic acid	C_8_H_6_O_4_	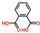	Phenolic acids	down (0/19167)	Xi
3-Hydroxyanthranilic acid	C_7_H_7_NO_3_	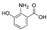	Alkaloid	down (0/461038)	Xn
3,4-Dihydroxybenzoic acid	C_7_H_6_O_4_	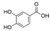	Phenolic acids	down (0/17677041)	Xi
3-O-Methylgallic acid	C_8_H_8_O_5_	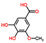	Phenolic acids	up	Xi
2,5-Dihydroxybenzoic acid	C_7_H_6_O_4_	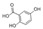	Phenolic acids	down (0.003 times)	Xi
5,7,3′,4′-Tetrahydroxyflavanone	C_15_H_12_O_6_	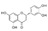	Flavonoids	down (0.003 times)	Xi
Dhurrin	C_14_H_17_NO_7_	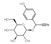	Alkaloid	down (0.004 times)	Xn
3-amino-2-naphthoic acid	C_11_H_9_NO_2_	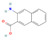	Alkaloid	down	Xn
2-Nitrophenol	C_6_H_5_NO_3_	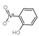	Phenolic acids	down	Xn
Histamine	C_5_H_9_N_3_	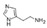	Alkaloid	down	Xn
Benzamide	C_7_H_7_NO	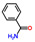	Alkaloid	down	Xn
Isoalantolactone	C_15_H_20_O_2_	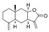	Terpenoids	down	Xn
N-benzylformamide	C_8_H_9_NO	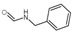	Alkaloid	down	Xn
3,4-Dihydrocoumarin	C_9_H_8_O_2_	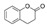	Lignans and coumarins	down	Xn
Genipin	C_11_H_14_O_5_	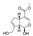	Terpenoids	down	Xn
Turgeniifolin A	C_19_H_18_O_6_	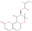	Lignans and coumarins	down	Xn

UAAQ, untreated *A. argyi* powder; BAAQ, boiled *A. argyi* powder. Xi, irritating substances. Xn, harmful substances.

## Data Availability

The original contributions presented in the study are included in the article/Supplementary Material; further inquiries can be directed to the corresponding author.

## Supplementary Materials

The following supporting information can be downloaded at: https://www.mdpi.com/article/10.3390/foods14183185/s1, Table S1: Sensory evaluation indicators and criteria; Table S2: Nutritional value per 100 g of BAAQ powder; Table S3: Weight gain, food intake and organ indexes in rats on day 14; Table S4: Hematological indexes of rats on day 14; Table S5: Serum biochemical indexes of rats on day 14.
