# Supplementary material for: Optimization of Its Preparation and Evaluation of Fresh and Tender Leaves of Artemisia argyi for Food Usage
Source: Foods. 2025 Sep 12;14(18):3185. doi: 10.3390/foods14183185 (PMC12469230; doi:10.3390/foods14183185)
Supplement: Supplementary file 1 [file foods-14-03185-s001.zip › foods-3821698-supplementary/foods-3821698-supplementary.pdf]

Table S1 Sensory evaluation indicators and criteria.

Indicator	Evaluation criteria
Color	Visual color comparison based on a standardized color card:<br>0: Very light yellow-green, no green dominance<br>1-2: Light yellow-green, yellow tone is dominant<br>3-4: Moderate yellow-green, yellow and green are balanced<br>5-6: Moderate green, true and clear green<br>7-8: Bright green, intense and vibrant<br>9-10: Deep green, very dark and saturated<br>Higher scores indicate a more intense and pure green color.
Aroma intensity	Purity and intensity of the characteristic aroma of *A. argyi:*<br>0: No perceptible aroma<br>1-2: Very faint, barely detectable<br>3-4: Light but identifiable herbal aroma<br>5-6: Moderate, clearly noticeable typical aroma<br>7-8: Strong, intense and pleasant aroma<br>9-10: Very strong, powerful and persistent typical aroma<br>Higher scores indicate more intense aroma.
Bitterness intensity	Intensity of bitter taste:<br>0: Extremely bitter, intolerable<br>1-2: Very strong bitterness, highly unpleasant<br>3-4: Strong bitterness, clearly unpleasant<br>5-6: Moderate bitterness, noticeable but acceptable<br>7-8: Mild bitterness, faintly perceptible<br>9-10: Very mild or no bitterness, imperceptible<br>Higher scores indicate less bitterness.
Bitterness persistence	Duration of bitter aftertaste:<br>0: No bitterness or immediate disappearance (0-5 seconds)<br>1-2: Very short persistence (6-15 seconds)<br>3-4: Short persistence (16-30 seconds)<br>5-6: Medium persistence (31-60 seconds)<br>7-8: Long persistence (1-2 minutes)<br>9-10: Very long persistence (2-3 minutes or more)<br>Higher scores indicate a longer persistence of bitterness.

Sensory evaluation was performed by a panel of 15 trained assessors. Assessments were conducted in individual sensory booths under controlled environmental conditions (e.g., white lighting, room temperature). Reference samples representing low, medium, and high intensity for each attribute were provided during training and evaluation to ensure consistency and accuracy in scoring.

Table S2 Nutritional value per 100 g of BAAQ powder.

Name	Amount	Name	Amount
Moisture	4.36 g	Copper	1.83 mg
Ash	6.76 g	Aluminum	3.56 mg
Protein	28.57 g	Zinc	6.01 mg
Fat	11.26 g	Iron	8.81 mg
Crude fiber	23.12 g	Calcium	1.71 g
β -carotene	2.93 mg	Potassium	305 mg
Vitamin C	7.99 mg	Sodium	841 mg
Vitamin B_2	1.95 mg	Manganese	44.4 mg
Selenium	3.5 μg	/	/

BAAQ, boiled tender leaves of *A. argyi* cv. qiai

Table S3 Weight gain, food intake and organ indexes in rats on day 14.

Item	Female			Male		
	FC	FUAAQ	FBAAQ	MC	MUAAQ	MBAAQ
Weight gain (g)	59.18 ± 7.01	67.12 ± 10.28	55.16 ± 14.44	83.63 ± 6.22[b]	89.93 ± 46.18[b]	102.65 ± 19.50[a]
Food intake (g/week)	100.72 ± 8.80[b]	141.35 ± 26.31[a]	117.51 ± 5.69[ab]	109.54 ± 6.14[b]	149.75 ± 21.57[ab]	158.49 ± 9.44[a]
Food titer (%)	19.51 ± 1.74[b]	23.97 ± 2.01[a]	23.39 ± 5.85[ab]	32.01 ± 4.69	29.02 ± 16.19	32.37 ± 5.64
Liver weight (g)	7.37 ± 0.82	7.98 ± 0.86	7.08 ± 0.67	9.07 ± 0.36	9.68 ± 1.49	9.99 ± 0.68
Liver index (%)	0.03 ± 0.00	0.32 ± 0.00	0.03 ± 0.00	0.03 ± 0.00	0.03 ± 0.00	0.03 ± 0.00
Spleen index (‱)	0.22 ± 0.03	0.24 ± 0.04	0.24 ± 0.36	0.26 ± 0.04	0.03 ± 0.28	0.25 ± 0.04
Kidney index (‱)	0.77 ± 0.09	0.76 ± 0.05	0.75 ± 0.05	0.84 ± 0.07	0.81 ± 0.08	0.83 ± 0.07
Testis index (‱)				0.96 ± 0.10	0.96 ± 0.10	0.91 ± 0.09
Epididymis index (‱)				0.21 ± 0.02	0.24 ± 0.03	0.24 ± 0.04
Epididymal fat index (‱)				0.77 ± 0.17	0.89 ± 0.22	0.78 ± 0.14

Mean values with different letters in the same row showed significant different within the same gender ($p<0.05$). Data are expressed as means ± SD, n = 10, except FUAAQ (n=7) and MUAAQ (n=9). FC, female control group; FUAAQ, female untreated *A. argyi* powder group; FBAAQ, female boiled *A. argyi* powder group; MC, male control group; MUAAQ, male untreated *A. argyi* powder group; MBAAQ, male boiled *A. argyi* powder group.

Table S4 Hematological indexes of rats on day 14.

Item	Female			Male		
	**FC**	**FUAAQ**	**FBAAQ**	**MC**	**MUAAQ**	**MBAAQ**
**WBC ($\times 10^9$/L)**	6.9 ± 2.55	5.1 ± 2.59	5.4 ± 0.63	6.6 ± 1.26	6.6 ± 1.37	8.2 ± 1.73
**RBC ($\times 10^{12}$/L)**	6.99 ± 0.39	6.77 ± 0.38	7.05 ± 0.22	6.79 ± 0.40^b	7.35 ± 0.28^a	7.41 ± 0.41^a
**HGB (g/L)**	141.9 ± 6.91	145.00 ± 8.58	147.60 ± 4.58	142.20 ± 6.58^b	150.67 ± 7.66^a	155.30 ± 5.85^a
**HCT (%)**	40.54 ± 1.88	41.93 ± 2.15	43.52 ± 1.56	42.61 ± 2.09^b	44.39 ± 1.98^{ab}	45.93 ± 1.72^a
**PLT ($\times 10^9$/L)**	1326.50 ± 136.71	1212.29 ± 144.02	1250.50 ± 143.34	1256.20 ± 111.13	1192.22 ± 253.29	1155.70 ± 80.74
**MPV (fL)**	7.82 ± 0.17	7.94 ± 0.29	7.85 ± 0.30	7.74 ± 0.29	8.10 ± 0.35	7.77 ± 0.25
**PCT (%)**	1.04 ± 0.11	0.96 ± 0.12	0.98 ± 0.12	0.97 ± 0.08	0.96 ± 0.18	0.90 ± 0.08
**MCV (fL)**	58.05 ± 2.43	61.96 ± 1.83	60.36 ± 1.82	62.90 ± 4.52	60.40 ± 2.26	62.08 ± 2.51
**MCH (pg)**	20.30 ± 0.61	21.41 ± 0.55	20.95 ± 0.54	21.00 ± 1.34	20.50 ± 0.72	21.00 ± 0.91
**MCHC (g/L)**	349.80 ± 5.55	345.71 ± 5.65	347.20 ± 3.26	334.00 ± 3.02	339.33 ± 4.72	338.10 ± 2.96
**NEUT (%)**	18.41 ± 2.33	22.96 ± 3.02	19.50 ± 3.49	26.56 ± 2.05	28.89 ± 11.58	25.71 ± 2.10
**LYMPH (%)**	80.15 ± 2.29	75.31 ± 2.04	75.73 ± 5.65	72.22 ± 3.12	70.12 ± 11.78	73.20 ± 3.23
**MONO (%)**	0.54 ± 0.23	0.69 ± 0.34	0.75 ± 0.40	0.72 ± 0.27	0.47 ± 0.30	0.42 ± 0.16
**EO (%)**	0.90 ± 0.56	1.04 ± 0.40	1.02 ± 0.42	0.50 ± 0.21	0.52 ± 0.28	0.66 ± 0.21
**BASO (%)**	0.00 ± 0.00	0.00 ± 0.00	0.00 ± 0.00	0.00 ± 0.00	0.00 ± 0.00	0.01 ± 0.03

Mean values with different letters in the same row showed significant different within the same gender ($p < 0.05$). Data are expressed as means ± SD, n = 10, except FUAAQ (n=7) and MUAAQ (n=9). WBC, White Blood Cell Count; RBC, Red Blood Cell Count; HGB, Hemoglobin; HCT, Hematocrit; PLT, Platelet Count; MPV, Mean Platelet Volume; PCT, Procalcitonin; MCV, Mean Corpuscular Volume; MCH, Mean Corpuscular Hemoglobin; MCHC, Mean Corpuscular Hemoglobin Concentration; NEUT, Neutrophils; LYMPH, Lymphocytes; MONO, Monocytes; EO, Eosinophils; BASO, Basophils. FC, female control group; FUAAQ, female untreated *A. argyi* powder group; FBAAQ, female boiled *A. argyi* powder group; MC, male control group; MUAAQ, male untreated *A. argyi* powder group; MBAAQ, male boiled *A. argyi* powder group.

Table S5 Serum biochemical indexes of rats on day 14.

Item	Female			Male		
	FC	FUAAQ	FBAAQ	MC	MUAAQ	MBAAQ
ALT (U/L)	31.30 ± 5.08	31.86 ± 8.80	31.90 ± 3.07	39.70 ± 5.81	38.89 ± 9.33	38.80 ± 4.94
AST (U/L)	108.60 ± 14.47[a]	90.14 ± 10.87[b]	98.80 ± 11.45[ab]	112.10 ± 13.92[a]	97.44 ± 12.02[b]	94.10 ± 17.58[b]
TP (g/L)	59.68 ± 2.68	61.49 ± 5.49	61.41 ± 2.59	55.43 ± 2.60[b]	56.78 ± 1.63[b]	58.94 ± 2.50[a]
ALB (g/L)	33.02 ± 1.46	33.91 ± 2.71	33.86 ± 1.82	30.23 ± 0.93[ab]	29.77 ± 1.48[b]	31.95 ± 1.03[a]
ALP (IU/L)	157.60 ± 36.79	147.14 ± 12.83	164.10 ± 22.28	209.20 ± 47.37	253.89 ± 90.15	249.40 ± 65.97
GGT (IU/L)	-0.21 ± 0.41	-0.11 ± 0.37	0.06 ± 0.22	-0.18 ± 0.32	-0.03 ± 0.21	0.14 ± 0.37
GLU (mmol/L)	6.78 ± 0.54[a]	6.01 ± 0.62[ab]	5.82 ± 0.63[b]	5.85 ± 0.80[b]	7.54 ± 1.34[a]	6.84 ± 0.95[a]
BUN (mmol/L)	3.92 ± 0.63	3.64 ± 0.91	3.66 ± 0.78	2.97 ± 0.82	3.52 ± 0.91	3.59 ± 0.59
CREA (μmol/L)	60.04 ± 4.43	55.90 ± 3.03	56.49 ± 3.54	48.80 ± 2.81	51.58 ± 3.85	51.86 ± 1.63
CHOL (mmol/L)	2.07 ± 0.32	2.23 ± 0.16	1.95 ± 0.35	2.06 ± 0.17	1.99 ± 0.14	2.13 ± 0.18
TG (mmol/L)	0.58 ± 0.12	0.57 ± 0.07	0.65 ± 0.27	0.79 ± 0.18	0.69 ± 0.25	0.88 ± 0.20
HDL-C (mmol/L)	1.34 ± 0.28	1.47 ± 0.13	1.27 ± 0.32	1.09 ± 0.16	1.04 ± 0.14	1.18 ± 0.14
LDL-C (mmol/L)	0.75 ± 0.19	0.87 ± 0.10	0.69 ± 0.22	0.80 ± 0.10	0.74 ± 0.08	0.85 ± 0.10

Mean values with different letters in the same row showed significant different within the same gender ($p < 0.05$). Data are expressed as means ± SD, n = 10, except FUAAQ (n=7) and MUAAQ (n=9). ALT, Alanine Aminotransferase; AST, Aspartate Aminotransferase; TP, Total Protein; ALB, Albumin; ALP, Alkaline Phosphatase; GGT, Gamma-Glutamyl Transferase; GLU, Glucose; BUN, Blood Urea Nitrogen; CREA, Creatinine; CHOL, Cholesterol; TG, Triglycerides; HDL-C, High-Density Lipoprotein Cholesterol; LDL-C, Low-Density Lipoprotein Cholesterol. FC, female control group; FUAAQ, female untreated *A. argyi* powder group; FBAAQ, female boiled *A. argyi* powder group; MC, male control group; MUAAQ, male untreated *A. argyi* powder group; MBAAQ, male boiled *A. argyi* powder group.
